# The transition from pediatric to adult care in individuals with Prader-Willi syndrome

**DOI:** 10.1530/EC-22-0373

**Published:** 2022-12-15

**Authors:** Christine Poitou, Anthony Holland, Charlotte Höybye, Laura C G de Graaff, Sandrine Bottius, Berit Otterlei, Maithé Tauber

**Affiliations:** 1 Assistance Publique-Hôpitaux de Paris, Centre de référence Maladies Rares (PRADORT, Syndrome de Prader-Willi et autres formes rares d’obésité avec troubles du comportement alimentaire), Service de Nutrition, Hôpital Pitié-Salpêtrière, Paris, France; 2 Department of Psychiatry, University of Cambridge, UK; 3 Department of Endocrinology and Department of Molecular Medicine and Surgery, Karolinska University Hospital and Karolinska Institute, Stockholm, Sweden; 4 Center for Adults with Rare Genetic Syndromes, Department of Internal Medicine, Division of Endocrinology, Erasmus Medical Center, University Medical Center Rotterdam, Rotterdam, The Netherlands; 5 Landsforeningen for Prader-Willis Syndrom Hiltonåsen, Slependen, Norway; 6 Centre de référence Maladies Rares (PRADORT, Syndrome de Prader-Willi et autres formes rares d’obésité avec troubles du comportement alimentaire), Service d’Endocrinologie, Obésités, Maladies Osseuses, Génétique et Gynécologie Médicale, Hôpital des Enfants, Toulouse, France

**Keywords:** Prader-Willi syndrome, transition

## Abstract

Prader–Willi syndrome (PWS), the most common form of syndromic obesity, is a complex neurodevelopmental genetic disorder including obesity with hyperphagia, endocrine and metabolic disorders and also psychiatric disorders. The most frequent endocrine disturbances include hypogonadism and growth hormone (GH) deficiency. Hypothyroidism and central adrenal insufficiency can also be observed but are less frequent. The transition of individuals with PWS from adolescence to adult life is challenging because of multiple comorbidities and complex disabilities. Individuals and caregivers face psychological, medical and social issues. This period of profound changes is thus prone to disruptions, and the main risks being the worsening of the medical situation and loss to follow-up of the individuals. Medical care may be poorly adapted to the needs of individuals because of a lack of knowledge concerning the syndrome and also lack of the necessary specific skills. A multidisciplinary panel composed of several experts in PWS met in November 2021 during an European Reference Network on Rare Endocrine Conditions (Endo-ERN) webinar. They presented complementary aspects of PWS from the perspective of the transition including psychiatric, pediatric and adult endocrinological and parent’s and patient’s points of view and shed light on the best way to approach this pivotal period.

## Introduction

Prader–Willi syndrome (PWS) is a complex neurodevelopmental genetic disorder including obesity consequent upon hyperphagia, psychiatric disturbances, endocrine and metabolic disorders (2). Global incidence is approximately 1 in 20,000 births in Europe. PWS is usually diagnosed early in life and then managed by pediatric teams.

Transition is defined as ‘a process of organized and coordinated change of adolescents and young adults with a chronic medical condition from a pediatric system of care to the adult system of care’(3). Transition is a process, whereas ‘transfer’ is the actual moment of the first appointment at adult care.

A European endocrinologist expert group in 2015 examined the gap in metabolic and endocrine care of individuals during transition (4). They highlighted the necessity of multidisciplinary collaboration across health sectors for endocrine diseases, the need for pediatric and adult endocrinologists to work together and early involvement of adolescents in their disease and also of parents in the transition process.

The transition of individuals with PWS from adolescence to adult life is particularly challenging for medical care not only because of multiple comorbidities and specific skills required for the medical team but also due to management of behavioral problems. This period of profound changes is thus prone to disruptions, the main risks being the worsening of the medical situation, breakdown in support and losing individuals to follow-up.

The Main Thematic Group 5 ‘growth and obesity’ of the Endo-ERN (https://endo-ern.eu/specific-expertise/growth-general-obesity-syndromes/) organized a webinar on November 16, 2021, and convened experts on the disease to address the transition of care in PWS. Speakers brought their knowledge concerning this critical period from psychiatric and endocrinological perspectives and also examined the complementary points of view of pediatricians and adult specialists. The experience of parents and individuals was also presented. This publication summarizes the presentations and discussions from this webinar.

## The psychiatrist's point of view

The transition from childhood to adulthood is a significant time in a person’s life and the life of their family. In some traditions, this transition is formally marked by a religious ceremony or a special celebratory occasion. It is a time of considerable change, ranging from biological to sociological. The move to greater independence is an expected part of life, bringing greater responsibilities and increasing freedom to make one’s own choices. For many people with complex disabilities associated with intellectual and cognitive impairments, such as people with PWS, transition can be a considerable challenge. There may be changes in living circumstances and also rates and severity of behavior problems may increase (5) and mental ill-health may develop for the first time (6). In such circumstances, a breakdown in support arrangements may occur with the consequences that transition is associated with a deterioration in wellbeing and quality of life.

Transition is about moving from what may be relatively protected circumstances in the family and in school to what is potentially a much more chaotic, demanding, complex and less-structured environment of adulthood. In adult life, the law places responsibility for decision-making on the individual. Society expects that adults take responsibility for their actions, and more advanced social, linguistic and cognitive skills are needed for individuals to function independently. International conventions and national laws rightfully place great importance on individual rights, which in adulthood means making one’s own choices, and respecting the principles of self-determination and autonomy. The UN Convention of the Rights of Persons with Disabilities (UN CRPD) (2006 UN General Assembly, *Convention on the Rights of Persons with Disabilities*, December 13, 2006, A/RES/61/106, Annex I, available at: https://www.refworld.org/docid/4680cd212.html) emphasizes that respect for these rights must be non-discriminatory and that they apply to everyone in society. Countries that have signed up to the UN CRPD are therefore expected to enable full and effective participation in society of people with disabilities. These policy and societal changes, which have taken place over the last 50 or more years, are very positive. However, what can be missing is the presence of the necessary services and skilled support to make the ideals of such legislation feasible and in a manner that minimizes the risk. This is the context in which transition takes place in many countries.

### Characteristics of PWS that impact transition

PWS is a condition that primarily affects the development of the nervous system, particularly the brain, resulting in regulatory and endocrine abnormalities of hypothalamic origin (2). Impaired development of cortical and sub-cortical areas of the brain (for review, see (7)) also results in particular neuropsychiatric and cognitive phenotypes characterized by intellectual and cognitive impairments, which particularly affect the higher order cognitive abilities of executive functioning, recognition of facial expression, task switching and planning (see review (8)).

One of the most striking aspects of the neuropsychiatric phenotype associated with PWS, which is related to impaired hypothalamic development, is the switch from under-eating and failure to thrive post natally, to the onset in early childhood of hyperphagia and an inability to self-regulate energy intake in line with energy expenditure (see review (9)). If access to food is not controlled at this time and throughout life, hyperphagia results in life-threatening obesity. Other aspects of the neuropsychiatric phenotype are increased propensity to major emotional outbursts, severe skin picking, the risk of affective disorder and particularly, in people with the chromosome 15 maternal uniparental disomy form of PWS, a high risk for developing an affective psychotic illness in late childhood and early adult life (see (10) for description of the neuropsychiatric phenotype).

Many of the physical and behavioral characteristics associated with PWS can be best conceptualized as a manifestation of impairments in specific physiological and psychological functions and associated behaviors, which have been shaped through evolution to maintain homeostasis and facilitate survival in what is inevitably demanding and changing physical and social environments. A particular challenge for people with PWS is therefore to be able to respond efficiently and effectively to the changes and demands of life. It is during transition that environmental demands are likely to be at their greatest, and it is at such times that higher-order cognitive and reasoning skills are required to plan and resolve any challenges. However, in late childhood and early adult life, the gap between the higher-order cognitive and functional abilities present in the typically developing population, necessary for adult life, and that of people with PWS, becomes even greater (sometimes referred to as the disability gap). At the time of transition, it is this combination of a less predictable and changing environment, on the one hand, and impairments in social cognition and other higher order cognitive abilities that may then lead to increased stress and anxiety and, for example, to emotional outbursts. Furthermore, such outbursts may become more problematic in the teenage years (5), partly as a consequence of the increasing height and physical strength of the person with PWS.

### Psychiatric co-morbidities associated with increasing age

Adolescence and early adult life in the typically developing population is the period when mood disorders and psychiatric illness, such as schizophrenia, may develop for the first time. In a systematic review of psychiatric illness in people with PWS, Aman *et al.* (under review) summarized the findings from 937 participants with PWS in 14 cohort studies and 25 case reports. The usual age of presentation of serious mental illness was in the teens and early adulthood, with as many as 60% of people with the maternal uniparental disomy form of PWS reported to have had such illnesses. These illnesses, characterized by sudden changes in mood, the onset of delusions and/or hallucinations, and other manifestations associated with atypical psychotic illness, most commonly have an acute onset and may be triggered by an infection or life stress (6). This increased risk of mental illness is around the age when transition is being planned or is taking place and the onset of such illnesses may go unrecognized as changes in mental state or behavior may simply be put down to transition taking place. In reality, the cause may be the onset of illness requiring diagnosis and effective treatment.

### What is needed?

There are no easy answers, but the risk of such transition-related problems can be reduced through understanding the challenges of transition for people with PWS by careful planning and most importantly, making the case for appropriate and informed support (see below). Some of the key issues to consider are summarized below.

1. A ‘rights-based’ approach, when preparing for adult life, is not simply about ensuring individual responsibilities and freedoms. It is also, importantly, about making the case for practical support (including access to funding) and the availability of services that are able to meet the particular needs of people with PWS at this time in their lives. This might include funding to access specialist PWS-friendly residential care or support to access adult education and special employment services that can meet the particular needs of people with PWS. It is informed support and access to services that can reduce the functional impact of the ‘disability gap’ described earlier.
2. In the younger generation of children with PWS, many appear to be more aware of the implications of having PWS and are fully engaged in preparing for change and in their plans for transition. To ensure a full understanding, information may be delivered using visual means so that it is better understood thereby reducing uncertainty. A national PWS association can be very helpful at this time for guidance, to make certain all those who are involved in transition are familiar with the specific challenges associated with PWS, most specifically the hyperphagia and the need for food security. Those involved in the transition process may or may not listen, but at the very least, they cannot argue that they were unaware of the issues. Murray *et al.* (11) examined case law regarding particular situations where some crisis or lack of services led to a legal challenge. Such case law is beginning to set important legal precedents in these countries in support of the rights of people with PWS to have services in the community according to their needs,
3. Be aware that this is the age in a person’s life when psychiatric illness may develop for the first time. These illnesses can be effectively treated if recognized and diagnosed, and with such treatments, mental state and behavior return to earlier levels. Access to appropriate mental health services that are willing and skilled enough to assess and treat mental illness in people with PWS is essential.
4. Allowing for some degree of risk, which may come with increasing freedom, may be appropriate, but always ensure that services are in place to respond if things deteriorate. What form such response takes will depend on whether or not the adult with PWS consents to the appropriate interventions. Laws that might help at such times will differ across countries. However, when someone with PWS enters into a downward spiral of increasing weight gain and a deterioration in behavior, some form of major intervention (e.g. hospitalization, residential hostels, psychiatric medications) is likely to be necessary. Working with the individual with PWS to achieve this is the ideal solution, but where a person’s decision-making capacity is impaired, the use of other legal means, with the appropriate protections and appeal mechanisms, may be appropriate. The challenge is the need to balance potential competing human rights: the right to life and the responsibility to act if life is at risk, for example, from severe obesity, on the one hand, and on the other, respect for the autonomy of a person who may have reached adulthood.

The availability of services and the ways that health and social care are funded vary globally. However, it is clear that people with PWS have complex needs, particularly relating to their behavior and mental health. In addition, at times such as in transition, where, for example, uncontrolled access to food may be allowed substantial weight gain will occur, resulting in physical consequences, such as diabetes mellitus and sleep apnea. Managing the transition to adult life with people with neurodevelopmental syndromes, such as PWS, touches on human rights, requires enlightened Government policies and access to local services and a health and social care workforce willing to inform themselves about the specific needs of people with PWS.

## The pediatrician’s point of view

Over the last 20 years, the diagnosis of PWS has been made in the first months of life (18 days in a recent French survey (1). The pediatric endocrinologist meets the family during the first month of life (1) and often coordinates the care of the child for about 18 years. Comprehensive and repeated evaluations of the child are organized to follow the nutritional, endocrinological and neurodevelopmental trajectories (Table 1) (1, 12, 13). The first months focus on improving feeding, interaction and communication, as the first post-natal phase of PWS is characterized by oral and social skill deficiencies (10, 11, 12, 13). Pediatric endocrinologists, dieticians, speech and language therapists, experts in infant feeding problems and physiotherapists are the most important professionals involved in early care for infants with PWS. Psychological support to parents is also required to help alleviate the burden of the recent diagnosis and the difficulties feeding and communicating with their baby. Growth hormone (GH) treatment is started during the first months of life generally around 6 months after performing polysomnography. A complete endocrine evaluation is done before the start of GH treatment and regularly repeated during follow-up (1, 2, 3, 4, 5, 6, 7, 8, 9, 10, 11, 12, 13, 14). Hyperphagia rarely starts before 6 years when adequate follow-up and support are set up. Scoliosis is regularly screened for during GH treatment, both during early childhood (14) and adolescence. Social and educational aspects are also part of the comprehensive care and follow-up, as is screening for psychiatric comorbidities. This is started as early as possible, to allow early identification of children and families who need specific support due to challenging behavior or social and economic deprivation.

**Table 1:** Comorbidities in Prader–Willi syndrome to be evaluated during the transition.

Endocrine features
Obesity related to hyperphagia is very common if not prevented
Growth hormone deficiency almost 100% in children and 50% in adults, SGA in 20%
Hypogonadism mixed > 60% (adult > 90%)
Cryptorchidism more than 90%
Premature or agressive adrenarche 30%
Hypothyroïdism 20–80%
Central adrenal insufficiency <5%
Metabolic
Diabetes about 25% started in adolescents associated with obesity
Dyslipidemia
Comorbidities
Scoliosis, sleep apnea, sleeping disorders ± narcolepsia ± catatonia
Gastro-intestinal problems, skin picking, autonomic dysfunction
Ophtalmology, dental issues
Psychomotor development
Speech and language problems, oral skills and communication issues
Cognitive evaluation, learning deficits
Behavior and psychiatric evaluation
Schooling orientation
Family and caregivers evaluation

Inducing puberty is necessary for most men with PWS, and many or most women with PWS undergo spontaneous breast development perhaps due to peripheral aromatization resulting in reasonably normal estrogen levels, although regular menstrual periods are rare without treatment. At completion of growth, a thorough evaluation of endocrine/metabolic issues, behavioral and other comorbidities is done in order to have a complete view of the adolescent and his/her family. This evaluation is often done by the pediatric team and it is a good opportunity to introduce healthcare professionals working in the adult team, such as the endocrinologist, psychologist, psychiatrist, ID physician and/or dietitian. The number of shared visits (with both the pediatric and adult endocrinologist), as well as the timing of the transfer to adult care, depends on the patient, the family and the local organization.

It is the moment, if not done before, to let the adolescent explain the project he/she imagines (expectations and wishes for their life including their job or occupation, socialization, their place and way of living, private and affective life) and, if possible, to try to make it real. As a whole, the care of the child and adolescent with PWS in the pediatric unit is the basis for a successful transition of care. Pediatric care focuses on early detection and management of comorbidities, prevention of obesity, support of the family, school and social orientation, prevention and/or care of severe behavioral troubles and prepares the transfer to adult care. Because PWS is so complex and changing with time and context, this multidisciplinary care requires (local or remote) coordination with expert centers.

Adolescence is a critical phase for all individuals but even more challenging for those with PWS as it is detailed in the last paragraph. Thus, adolescence and transition of care should be well prepared. Topics like guardianship legal representation, sexuality, vulnerability and other important issues should be addressed in collaboration with parents and caregivers. There should also be room for psychosocial support: families may be exhausted and are often anxious regarding the transition to adulthood. Adolescents with PWS feel they are different, but they often want to be like others. They often have a strong desire to lead a normal life with love and romantic thoughts about raising a family (2). Adolescents, young adults, caregivers and relatives usually have questions regarding autonomy and social and sexual relationships. Unfortunately living in a total autonomy is often not possible, due to hyperphagia, intellectual disability or other PWS-related problems that may require support. Although pregnancies have been described, fertility is extremely rare for women and not reported in men with PWS. However, contraception should be discussed if the woman has ovarian activity and is sexually active without barrier methods (15).

Taken together, optimal pediatric care, careful coordination and trust between individuals, relatives, caregivers and the (pediatric and adult) healthcare professionals are crucial for proper transition.

## The adult endocrinologist's point of view

### Growth hormone treatment

GH treatment is for more than three decades a well-established and approved treatment in children with PWS in many countries (2, 16, 17). In contrast, PWS is an approved indication for GH treatment in the transition period or for adults only in a few countries (2, 17). Therefore, in most countries, the diagnosis of adult GH deficiency must be established according to guidelines before it can be used in the transition period and in adults with PWS.

The transition period raises question about continued GH treatment and treatment outcomes after final height has been reached. As mentioned previously, pediatric services are usually functioning very well with a well-established multidisciplinary team. In contrast, adult services are less developed and the number of adult PWS clinics is insufficient – in many places, there is no team. PWS is a complex, multisystem disease (2). Cognitive and behavior disabilities and obesity and hormone deficiencies are frequently present, and they can all affect GH secretion (2, 16, 17).

GH secretion peaks at puberty, after which GH secretion gradually decreases with age. In children, GH is important for skeletal growth and, in both children and adults, GH has important metabolic effects; it increases muscle mass and decreases fat mass, increases metabolism, has beneficial effects on blood lipids, increases blood glucose and improves quality of life (QoL).

It has been established that GH secretion is insufficient in children with PWS, whereas insufficient GH secretion in adults with PWS has been debated. Nevertheless, there are several arguments for a persisting GH deficiency beyond childhood in PWS. PWS is a chronic hypothalamic disease and hormone deficiencies seen in children with PWS that would also be expected to be seen in adults with PWS (2, 16). GH stimulation tests in adults with PWS have shown GH deficiency in 0–67% (2, 16, 18), but the GH deficiency is of hypothalamic origin and most GH stimulation tests stimulate the secretion of GH from the pituitary gland which may result in falsely normal GH responses (2, 16). The marker of GH activity insulin-like growth factor I (IGF-I) is below normal in 75–91% (2, 16). However, there is a large overlap between IGF-I levels in individuals with GH deficiency and levels in healthy controls and IGF-I are affected by nutritional status and hypogonadism (2, 16). There might also be differences in GH molecular weight, defects in the GH receptor or insufficient spontaneous GH secretion, which might have clinical consequences similar to GH deficiency. This has not been fully elucidated. Furthermore, PWS is a multi-system disease and different interpretations of tests and examinations are required compared to a typical GH-deficient patient. Finally, several studies have consistently shown significant benefits of GH treatment on growth, body composition and physical and psychosocial function in both children and adults with PWS (19).

Thus, there are several studies documenting GH’s positive effects on body composition in adults with PWS as well as some in the transition period (19, 20, 21, 22).

Other studies have examined the effect of GH treatment on physical capacity in adults with PWS (2, 16, 17) and on bone mineral density (2, 16, 17, 23) (24, 25).

Evaluation of GH’s effect on QoL in PWS is difficult and few studies have been published (2, 16). Improvements in measures of neuropsychological function as well as QoL were repeatedly reported (2, 16, 17).

No compliance or adherence problems or major safety concerns were reported in studies or in real life during the transition period or in adulthood. Few and expected side effects to GH treatment occurred. They included transient and dose-dependent headache, nausea, edema and joint pain (2, 17, 19, 26). Also, as expected, a small increase in fasting glucose and trends toward higher insulin and insulin resistance was seen (26). Increase in diabetes has not been observed during GH treatment (2, 16, 19, 26).

No clinically significant negative impact of long-term GH treatment on respiratory or sleep parameters in adults with PWS was observed (27, 28), and available studies have not shown that GH treatment negatively affects the onset or progression of scoliosis even after long-term treatment (2, 16, 29).

PWS is associated with GH deficiency in all ages. GH treatment has many beneficial effects and few side effects and should be continued during the transition period and in adulthood in the absence of contraindications. However, GH treatment in the transition period and in adults is only approved in a few countries, and the high costs of GH and lack of reimbursement might prohibit its continued use in many countries. Therefore, efforts are needed to increase perception of the benefits and to prioritize continuous GH treatment in PWS after final height has been reached (17).

### Adrenal insufficiency and hypogonadism

PWS is associated with pituitary hormone deficiencies. Two types of hormone deficiencies are especially crucial during the transition to adulthood, as their treatment differs greatly between children and adults.

The first relevant deficiency is central adrenal deficiency (CAI). There is no consensus on the prevalence of CAI in adults with PWS and the need for hydrocortisone administration in adults with PWS is unclear. This is due to the use of different dynamic function tests to diagnose CAI and to the fact that most studies involved children instead of adults (30, 31, 32, 33, 34, 35, 36, 37, 38). In some countries, it is general practice to administer hydrocortisone during stressful situations, such as surgery, illness or intense psychological stress (30, 31). However, side effects of frequent use of hydrocortisone are weight gain, diabetes mellitus, hypertension and osteoporosis (39), already major health issues in adults with PWS (40). As both under- and overtreatment with hydrocortisone can cause severe problems for the patient, it is important to know the true prevalence of CAI in adults with PWS. Therefore, PWS experts from the International Network for Research, Management and Education on adults with PWS (INfoRMEd-PWS) have collaborated to define the prevalence of CAI in adults with PWS (41). We conclude that CAI is very rare (1.2%) in adults with PWS. In order to prevent overtreatment with hydrocortisone, we advise *against* routine hydrocortisone administration during psychological stress, illness or surgery in adults with PWS. In individuals in whom there is a significant clinical suspicion of hypocortisolism (such as apathy, fainting or observed hypotension during acute infections or other stressful events), we recommend testing to exclude CAI and only administer hydrocortisone if CAI is confirmed by ITT or (s)MTP.

The other hormone deficiency that is especially important during the transition phase is hypogonadism. Untreated hypogonadism can cause osteoporosis at older adult age, which is already an important issue in PWS. Therefore, early detection and treatment of hypogonadism are crucial. In order to prevent undertreatment, we retrospectively collected data on physical examination, biochemical measurements and sex hormone replacement therapy (SHRT). During the MTG5 webinar, we shared our experience with hypogonadism in a Dutch adult PWS cohort and we have shown the results of our retrospective study. Hypogonadism was present in 98% of males and 94% of females. Both primary and central hypogonadism were present, as well as mixed forms. The major reason why individuals did not receive (adequate doses of) SHRT was for behavioral challenges.

Based on our experience, an expert panel discussion of members of the INfoRMEd-PWS network and a thorough review of the literature, we provide practical algorithm for SHRT in males and females (15, 42) in order to prevent undertreatment and the associated adverse health outcomes.

## Management of transitional care in PWS: the central role of the coordination

As we mentioned before, transition in PWS is challenging because of emergence or worsening of many comorbidities such as hyperphagia, behavioral problems and obesity-related complications, in particular, type 2 diabetes.

We conducted a retrospective study in a cohort of 95 adults with PWS to evaluate and compare both endocrine and metabolic parameters depending on the transition status of those who received transition care and those who did not (43). We highlighted that endocrine management during the transition period was made difficult by multiple comorbidities, especially psychiatric disorders. We also showed that a coordinated care pathway with specialized pediatric care and organized transition from a pediatric hospital to a Reference Center for adults with PWS with multidisciplinary follow-up involving health and social care professionals contributed to better metabolic and psychological health in adulthood. We recommended systematically performing a complete endocrine and neuropsychiatric assessment at the end of growth, in the pediatric unit before referring individuals to an adult department, to improve endocrine management. Finally, we emphasized that to be effective, the transition should not only focus on endocrine aspects but also consider social, psychiatric and nutritional issues.

**Figure 1:**
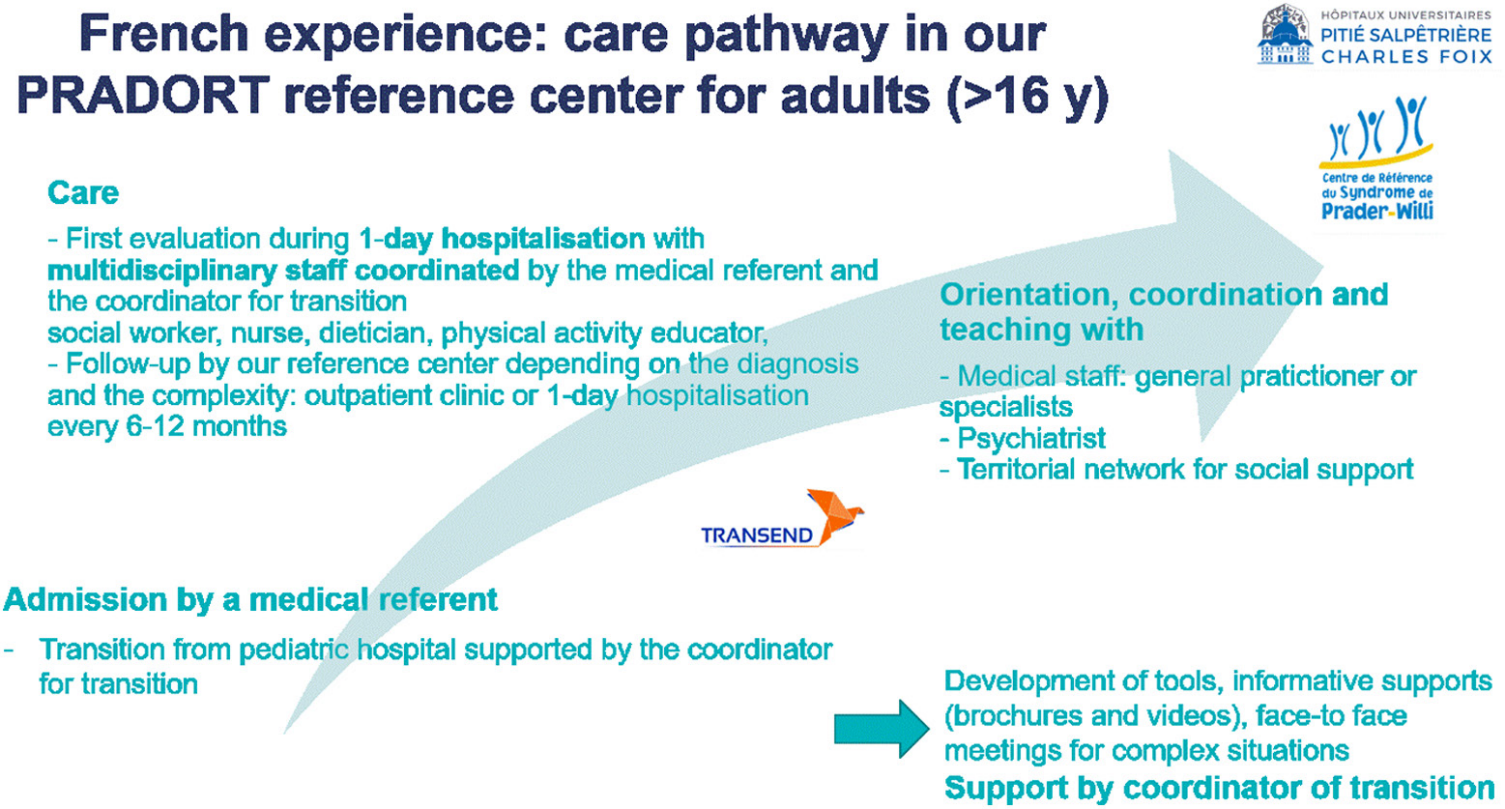
Comprehensive care of young adult in a French reference center. All the dimensions are evaluated with a global multidisciplinary approach including endocrine and obesity-related comorbidities, behavioral and psychological dimensions, psychosocial impact on the patient and the caregiver, social support.

A well-organized transition requires a multidisciplinary team to manage the syndrome as a whole, supervised by a specialist for adults with PWS, following early and structured care in a pediatric department. The transition should be supported medically by endocrinologist/diabetologist/nutritionist and experienced multidisciplinary teams with psychological/psychiatric expertise, in pediatrics and then in adult services. As an example, we show here the organization in our French reference center (Fig. 1).

As the transition period is crucial for individuals with chronic diseases and more particularly in the case of PWS, it is important to anticipate this period by coordinating with the different actors. Indeed, in addition to medical care, the transition is multifaceted: social integration (relationships, living space), activities and leisure, employment, protection of the young person, etc.

In this context, the role of a coordinator of transition is particularly important. Sandrine Bottius, the healthcare pathway coordinator of the transition program *Transend*, Pitié-Salpêtrière hospital (Paris), gives her experience with young people with PWS in a video visible on the website (*https://www.youtube.com/watch?v=Tikny0mZQxg&t=53s*). Briefly, her role is to ease the transition between pediatric and adult hospitals. She represents a resource person for the young person and his or her relatives in the hospital. She ensures the continuity of the care pathway and contributes also to the link between the intra and extra hospital sectors. She emphasized that people with PWS present specific characteristics that must be considered. For example, they need stable landmarks and reassurance. The rigidity of thoughts, the different levels of intellectual disabilities and social impairment require adaptation of communication and support materials to make them accessible to young adults with PWS and to their caregivers. Finally, in this video, she gives practical examples of her action as coordinator of the healthcare pathway for individuals with PWS in transition.

## The parent’s view: ‘thank you for listening’

### Background

I am a mother of a young woman who is 23 years with PWS, living in Norway. My daughter was diagnosed at 1 month with the deletion type of PWS. We used a feeding tube for 3 months, and she walked when she was 3 years old and started using GH at 7 years of age. She has classical PWS features, gastro-esophageal reflux and a mild cognitive disability. But she has never been overweight because she had an early diagnosis and care and we could maintain food control.

### Dealing with PWS from childhood to adolescence

At the physical level, the puberty started normally but stopped at a point, and around 15 years, we convinced the doctor to give her some hormones to ‘pull through’ and this resulted in her starting menstruation. She used hormones for about 1 year, and after that, everything was working out fine. The reason why we engaged in this was a lecture from a medical expert at an International Prader–Willi Syndrome Organization congress where he said that we should give young persons with PWS the opportunity to have as normal transition as possible. In addition to the menstruation, we also experienced that her body composition developed into more woman-like features. Our experience is that it was important for her to be able to feel like other girls in school and also as her older sister.

At the psychological level, we have tried to prepare her for adolescence in several ways. One big issue discussed, which was hard but necessary, was that she would be unable to be a mother. We talked to her about that when she was only 9 years old. Only weeks later, she was confronted by this at school because someone has read about it on internet – I was so glad that we were able to be in front of that.

We have also tried to teach her to be as independent as possible, as we knew that many areas of her life would be restricted.

She now lives in an apartment with 24/7 assistance. That is something we have been preparing here for all her life, as we have with her siblings; we have to notice that in Norway, it is most common to move out sometime between 20 and 25.

During the transition, we have experienced periods that have been extra challenging. Two such periods were very much alike with the same symptoms but very different outcomes.

The symptoms were depression, anxiety, almost paranoid thoughts and a low emotional sensibility. In both situations, we did a thorough ‘investigation’ into her environment at home, at school and workplace and among her social connections. In one of the situations, we found a lot of uncertainties, turnover among the staff and bad communication. When we were able to change that, everything changed for her and the symptoms went away. In the other situation, we tried the same but could not find obvious reasons. Luckily, she had an appointment with her endocrinologist at that time and she found very good reasons for her symptoms: a disturbed metabolism that leads to the diagnosis of hyperthyroidism (Graves’ disease). My daughter never complained about the usual symptoms of this disease, but when she started using medication, she stated: *now it is gone!* She experienced several symptoms but was not able to explain them to us. That was the same difficulty when she was diagnosed with reflux. So, our learning has been*: never trust the symptoms – or lack of those!*

In summary, what has been most important for us, parents to someone with PWS?

1. Knowledge about PWS and what is coming up next. This knowledge has been possible thanks to other parents in our national PWS organization, from our national resource center Frambu and from IPSWO.
2. The knowledge of ‘*where to go with what’*.
3. Doctors who are listening! In Norway, we almost never meet doctors who have met many other people with PWS, so it is really important that they are listening to what we know about PWS or where to find the right information.

Our wishes for the future are:

1. Every family with PWS is connected to other PWS families.
2. Clear healthcare pathways for PWS in each country with the possibility for every person with PWS to meet a doctor who has already met many others with PWS.
3. Clear guidelines should include both the health system, the social care system, schools and workplace.

A big thank you to all doctors who have been listening to us and to all doctors who are listening to the person with PWS and to their families. My experience is that those doctors who are listening, are also acting, and acting creates possibilities. THANK YOU!

## Conclusion

During transition, the care must be comprehensive with close collaboration between reference centers for PWS and the multidisciplinary specialists involved (endocrinologist and nutritionist, psychiatrist, etc.).

Transition in people with PWS is impacted by two parallel factors common to all people with PWS regardless of genetic type: first, atypical brain development and the associated social, cognitive and functional impairments and, secondly, a transition environment characterized by increasingly complex demands and the impact of greater independence and by unpredictability and uncertainty. It is the interactions between the biological and the psychological and the environmental that can give rise to major difficulties. For people with uniparental disomy, in particular, there is also the risk that a serious co-morbid psychiatric illness may develop.

This vulnerable period needs to work on the environment and extended support. Coordination between the caregivers and social actors is essential to improve the care pathways and daily life of individuals, which are very strongly intertwined. Actors involved in these pathways have a crucial role to help the families.

## Declaration of interest

AH has been advisor to a number of pharmaceutical companies and he is President of the International PWS Organisation. CP has been advisor for Rhythm and Novo Nordisk companies. CH has received lecture fees from Novo Nordisk, Sandoz and Pfizer and has consulted for Novo Nordisk Scandinavia.

## Funding

This publication has been supported by Endo-ERN, which is co-funded by the European Union’s 3rd Health Programme (CHAFEA Framework Partnership Agreement No 739527).

## Author contribution statement

AH wrote the section ‘The psychiatrist’s point of view’, MT the section ‘The pediatrician’s point of view’, CH the section ‘The adult endocrinologist’s point of view’, LdG the section ‘Management of transitional care in PWS: the central role of the coordination’, CP and SB the section ‘The parent’s view: ‘Thanks for listening’, BO the section ‘Conclusion’. CP and MT coordinated the work.
